# Irisin stimulates protective signaling pathways in rat hippocampal neurons

**DOI:** 10.3389/fncel.2022.953991

**Published:** 2022-09-09

**Authors:** Mychael V. Lourenco, Guilherme B. de Freitas, Ícaro Raony, Sergio T. Ferreira, Fernanda G. De Felice

**Affiliations:** ^1^Institute of Medical Biochemistry Leopoldo de Meis, Federal University of Rio de Janeiro, Rio de Janeiro, Brazil; ^2^Department of Biomedical and Molecular Sciences, Centre for Neuroscience Studies, Queen’s University, Kingston, ON, Canada; ^3^Department of Psychiatry, Centre for Neuroscience Studies, Queen’s University, Kingston, ON, Canada; ^4^Institute of Biophysics Carlos Chagas Filho, Federal University of Rio de Janeiro, Rio de Janeiro, Brazil; ^5^D’Or Institute for Research and Education, Rio de Janeiro, Brazil

**Keywords:** FNDC5/irisin, Alzheimer’s disease, hippocampus, reactive oxygen species (ROS), gene expression

## Abstract

Physical exercise stimulates neuroprotective pathways, has pro-cognitive actions, and alleviates memory impairment in Alzheimer’s disease (AD). Irisin is an exercise-linked hormone produced by cleavage of fibronectin type III domain containing protein 5 (FNDC5) in skeletal muscle, brain and other tissues. Irisin was recently shown to mediate the brain benefits of exercise in AD mouse models. Here, we sought to obtain insight into the neuroprotective actions of irisin. We demonstrate that adenoviral-mediated expression of irisin promotes extracellular brain derived neurotrophic factor (BDNF) accumulation in hippocampal cultures. We further show that irisin stimulates transient activation of extracellular signal-regulated kinase 1/2 (ERK 1/2), and prevents amyloid-β oligomer-induced oxidative stress in primary hippocampal neurons. Finally, analysis of RNA sequencing (RNAseq) datasets shows a trend of reduction of hippocampal FNDC5 mRNA with aging and tau pathology in humans. Results indicate that irisin activates protective pathways in hippocampal neurons and further support the notion that stimulation of irisin signaling in the brain may be beneficial in AD.

## Introduction

Irisin is an exercise-induced myokine that modulates adipose, bone and brain functions (Boström et al., 2012; Kim et al., 2018; De Freitas et al., 2020; Isaac et al., 2021; Jodeiri Farshbaf and Alvina, 2021). Upon exercise, irisin is cleaved from a precursor protein, fibronectin type III domain containing protein 5 (FNDC5), and released into the circulation (Boström et al., 2012; Jedrychowski et al., 2015). FNDC5 and irisin have been detected in the brain (Wrann et al., 2013; Lourenco et al., 2019), and irisin has been shown to mediate the beneficial actions of physical exercise in mouse models of Alzheimer’s disease (AD) (Lourenco et al., 2019).

Previous studies have identified potential signaling mechanisms induced by irisin in the brain. Wrann et al. (2013) reported that irisin stimulates the expression of brain-derived neurotrophic factor (BDNF) in cultured neurons (Wrann et al., 2013), and we have demonstrated that irisin engenders cAMP/PKA/pCREB signaling in brain explants (Lourenco et al., 2019). Recently, αVβ5 integrin has been suggested as a potential receptor for irisin in the brain (Islam et al., 2021). However, the signaling mechanisms initiated by irisin in the brain are not completely understood. Here, we used primary hippocampal cultures to gain insight into potential irisin-mediated neuroprotective pathways.

## Methods

### Primary hippocampal cultures

Cultures were prepared in compliance with international standards. Experiments were approved by the Institutional Animal Care and Use Committee of the Federal University of Rio de Janeiro (protocol #IBqM 022). Primary rat hippocampal neuronal cultures were prepared according to established procedures (Bomfim et al., 2012; Lourenco et al., 2013), maintained in Neurobasal medium supplemented with 2% B27 (Life Technologies, CA), 1 mM glutamine, penicillin/streptomycin, and amphotericin B (De Felice et al., 2007), and were used after 18–21 days *in vitro*. Cultures were exposed to recombinant irisin (25 nM) (Adipogen, Switzerland) for the time intervals indicated in each experiment. Choice of this concentration was based on previous studies showing neuroactive properties of 25 nM irisin, in the absence of any detectable toxicity (Moon et al., 2013; Peng et al., 2017; Lourenco et al., 2019). For ROS experiments, 0.5 μM Aβ oligomers (AβOs) or an equivalent volume of vehicle (2% DMSO in PBS) were added 15 min after irisin and remained for 3 h. Adenoviral vectors expressing FNDC5 or GFP (MOI 1) [as described in Lourenco et al. (2019)] were allowed to express for 48 h before conditioned medium was collected from primary neurons. For some experiments, hippocampal cultures were exposed to forskolin (10 μM) or an equivalent volume of vehicle (0,1% ethanol) for 20 min.

### ELISA

Conditioned medium was collected, centrifuged at 10,000 × *g* for 10 min at 4°C, and the supernatant supplemented with protease and phosphatase inhibitor cocktails. Irisin (Phoenix; EK-067-29) and BDNF (Abcam; ab99978) ELISA assays were performed according to kit manufacturer instructions, as previously described (Lourenco et al., 2019, 2020).

### RNA extraction and qPCR

Total RNA was obtained from cultures using the SV Total RNA Isolation System (Promega, CA, United States), following manufacturer instructions. Purity and integrity of extracted RNA were checked by the 260/280 absorbance ratio. Only preparations with 260/280 nm optical density ratios higher than 1.8 were used. RNA concentrations were determined by absorption at 260 nm. For qRT-PCR, 1 μg total RNA was used for complementary DNA synthesis using High Capacity cDNA Reverse Transcription kit (Applied Biosystems, CA, United States). Quantitative expression analysis of target genes was performed on a 7500 Applied Biosystems (Foster City, CA, United States) system with the Power SYBR Green kit, as described (Lourenco et al., 2019; De Bastiani et al., 2022; Raony et al., 2022). β-actin (*actb*) was used as an endogenous reference gene for data normalization. qRT-PCR was performed in 15 μL reaction volumes. Primer sequences used in this study were: rat bdnf (Fw: CACTGAAGGCGTGCGAGTATT; Rv: TGTACTCCTGTTCTTAGCAAA) and rat actb (fw: TACTGCCCTGGCTCCTAGCA; Rv: TCAGGAGGAGCAA TGATCTTGAT). Cycle threshold (Ct) values were used to calculate fold changes in gene expression using the 2^–ΔΔCt^ method (Livak and Schmittgen, 2001).

### Immunoblotting

Hippocampal cultures were homogenized in RIPA buffer containing protease and phosphatase inhibitor cocktails and were resolved on 4–20% polyacrylamide pre-cast gels (BioRad) with Tris/glycine/SDS buffer run at 150 V for 60 min at room temperature. The gel (30 μg total protein/lane) was electroblotted onto Hybond ECL nitrocellulose using 25 mM Tris, 192 mM glycine, 20% (v/v) methanol, pH 8.3, at 300 mA for 90 min at 4°C. Membranes were blocked with 3% bovine serum albumin (BSA) for 1 h at room temperature, as described (Freitas-Correa et al., 2013). Primary antibodies anti-pERK 1/2 (Cell Signaling, 4377S; 1:1000), anti-total ERK 1/2 (Cell Signaling, 9102S; 1:1000), and anti-β-actin (Abcam, ab170325; 1:20,000) were diluted in blocking solution and incubated with the membranes overnight at 4°CC. After incubation with anti-mouse or anti-rabbit fluorescent IRDye-conjugated secondary antibodies (1:10,000) for 60 min, membranes were washed and then scanned in an Odyssey detector. Optical density determination for quantification was performed on ImageJ (Abràmoff et al., 2004).

### Amyloid-β oligomers

Oligomers were prepared from synthetic Aβ_1–42_ (California Peptide) and were routinely characterized by size-exclusion chromatography, as described (De Felice et al., 2007; Sebollela et al., 2012; Oliveira et al., 2021).

### Reactive oxygen species assays

Reactive oxygen species formation was evaluated in living neurons using CM-H_2_DCFDA as described previously (De Felice et al., 2007; Saraiva et al., 2010; Brito-Moreira et al., 2017). Briefly, 2 μM CM-H_2_DCFDA was loaded during the last 40 min of AβO exposure. Neurons were rinsed three times in warm PBS containing 2% glucose and immediately imaged on a Nikon Eclipse TE300 inverted microscope. Analysis of DCF fluorescence was carried out using ImageJ. Ten images were acquired under each experimental condition, carried out in triplicate, per experiment. Each experiment was performed with independent primary cultures.

### Human data sets

RNA-seq data was obtained from The Aging, Dementia and Traumatic Brain Injury Study,^1^ a detailed transcriptomics and neuropathological investigation of the aging human brain. Inclusion criteria encompassed subjects (males and females) >77 years old and complete information of hippocampal Fndc5 gene expression and neuropathology. Exclusion criteria were presence of non-Alzheimer’s dementia, mixed pathologies, or previous traumatic brain injury (TBI) diagnosis. Differential gene expression was based on comparison of normalized z-scores obtained from the study. For Braak analyses (Braak and Braak, 1991), subjects were classified as either low tau pathology (Braak I–II) or high tau pathology (Braak III–VI). Detailed information of tissue processing, neuropathological analyses, RNA extraction and deep sequencing can be found online (see text footnote 1). Complete datasets can be downloaded from this link: https://aging.brain-map.org/download/index.

### Statistical analysis

Data are expressed as means ± S.E.M. and were analyzed using GraphPad Prism 6 software (La Jolla, CA, United States). Data were assessed for normality using the Shapiro–Wilk test prior to statistical comparisons. For normally distributed data, comparisons between multiple experimental groups were analyzed using two-tailed ANOVA, followed by appropriate *post-hoc* tests. Comparisons between two groups were analyzed by two-tailed Student’s *t*-test. For comparisons between two groups deviating from normality, Wilcoxon matched-pairs rank test was used. Correlations between hippocampal FNDC5 expression and markers of AD-related neuropathology (Aβ and tau) from human datasets were evaluated by Pearson (parametric) or Spearman (non-parametric) correlation analyses. Sample sizes and *p*-values for each experiment are indicated in the Figure.

## Results and discussion

Building upon previous evidence linking irisin to BDNF in rodents and humans (Wrann et al., 2013; Lourenco et al., 2020), we initially treated primary rat hippocampal cultures with recombinant irisin (25 nM) for 24 h and determined BDNF mRNA levels. We found that irisin-treated cultures had increased BDNF mRNA content as compared to vehicle-treated cultures (Veh: 1.0 ± 0.15; Irisin: 10.6 ± 5.2) (Supplementary Figure 1).

**FIGURE 1:**
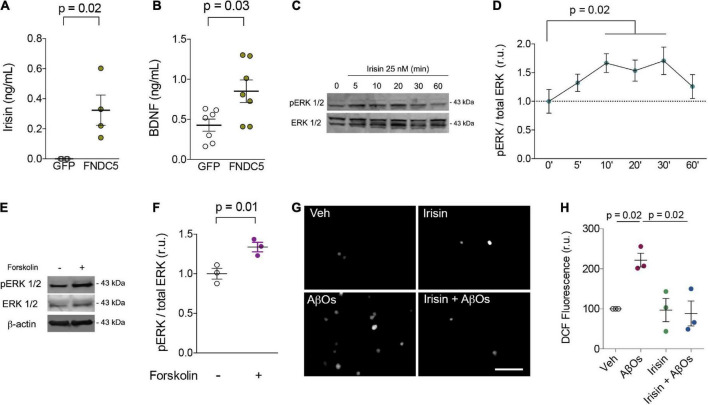
Irisin increases extracellular BDNF levels, stimulates transient ERK activation, prevents AβO-induced oxidative stress in primary neurons. **(A,B)** Primary hippocampal cultures were transduced with AdFNDC5 or AdGFP (control) adenoviral particles for 48 h. Conditioned media were collected and levels of irisin **(A)** or BDNF **(B)** were assessed by ELISA. *N* = 4 experiments using independent hippocampal cultures for irisin and 7 for BDNF measurements. Wilcoxon matched-pairs rank test. Primary hippocampal cultures were treated with recombinant irisin (25 nM) for the indicated timespoints **(C,D)** or with forskolin (10 μM; 20 min) **(E,F)**, and ERK 1/2 phosphorylation at Thr202/Tyr204 (pERK 1/2) was measured by immunoblotting. *N* = 3 experiments using independent hippocampal cultures. Repeated measures one-way ANOVA with Holm-Sidak correction. **(G,H)** Hippocampal neurons were exposed to 0.5 μM AβOs for 3 h in the presence or absence of recombinant irisin (25 nM). When present, irisin was added 15 min before AβOs. ROS was measured by DCF fluorescence. *N* = 3 experiments with independent cultures and AβO preparations. Two-tailed two-way ANOVA with Holm-Sidak correction. Each dot represents an independent hippocampal culture; data are shown as means ± S.E.M. *p*-values are indicated in the figure. Scale bar = 100 μm.

Primary rat hippocampal cultures were next transduced with adenoviral vectors to overexpress FNDC5 (AdFNDC5) or GFP (AdGFP, as a control) for 48 h. Whereas control media had undetectable levels of irisin, conditioned media from AdFNDC5-transduced cultures contained high amounts of soluble irisin (0.32 ± 0.1 ng/ml) (Figure 1A). Notably, expression of FNDC5/irisin by AdFNDC5 resulted in an increase in extracellular BDNF when compared to cultures transduced with AdGFP (GFP: 0.43 ± 0.08 ng/ml; FNDC5: 0.85 ± 0.14 ng/ml; *W* = 24; *p* = 0.03) (Figure 1B). These results indicate that extracellular BDNF content is potentially increased by irisin, likely contributing to synapse function and neuronal homeostasis.

Irisin has been reported to engage extracellular signal-regulated kinase 1/2 (ERK 1/2) signaling to promote adipose tissue browning (Zhang et al., 2014). In neurons, ERK 1/2 has been associated with neuroprotective mechanisms induced by BDNF against toxic insults (Almeida et al., 2005). Treatment with recombinant irisin (25 nM) transiently (from 10 to 30 min) promoted ERK 1/2 phosphorylation at threonine202/tyrosine204 residues (Thr202/Tyr204) in hippocampal neurons, indicative of ERK activation (Figures 1C,D). Interestingly, treatment with forskolin, a direct activator of adenylyl cyclase, for 20 min similarly triggered ERK 1/2 phosphorylation in primary neurons (Veh: 1 ± 0.07; Forskolin: 1.34 ± 0.06; *t* = 12.39; *p* = 0.01) (Figures 1E,F). Results indicate that the stimulation of cAMP signaling by irisin (Lourenco et al., 2019) is linked to ERK activation to amplify protective responses and prevent neuronal dysfunction. Whereas chronic ERK activation has been reported in mouse models of AD (Kirouac et al., 2017), it is conceivable that moderate/transient ERK phosphorylation may result in neuroprotection in AD.

We next tested whether irisin would protect cultured hippocampal neurons against oxidative stress induced by AD-linked amyloid-β oligomers (AβOs) (De Felice et al., 2007; Brito-Moreira et al., 2017). When added to cultures 15 min before AβOs, recombinant irisin (25 nM) prevented AβO-induced accumulation of reactive oxygen species (ROS), as measured by dichlorofluorescein (DCF) fluorescence (Veh: 100; AβOs: 221 ± 17; Irisin: 97 ± 29; Irisin + AβOs: 88 ± 31; two-way ANOVA interaction *p* = 0.02) (Figures 1G,H). These results are consistent with the kinetics of irisin-induced protective mechanisms and demonstrate that irisin mitigates neuronal oxidative stress, a hallmark of metabolic dysfunction in AD pathogenesis (Smith et al., 1998; De Felice et al., 2007; Clarke et al., 2018). Notably, recent studies demonstrated that irisin prevents oxidative stress in cardiomyocytes and endothelial cells (Zhang et al., 2020; Lin et al., 2021; Pan et al., 2021), raising the notion that mitigation of oxidative stress may be a shared mechanism of protection induced by irisin in the brain and in other tissues. Altogether, our results support the notion that boosting brain irisin levels (achieved by regular physical exercise, for example) could entail a neuroprotective mechanism that alleviates the impact of neuronal injury in AD.

**FIGURE 2:**
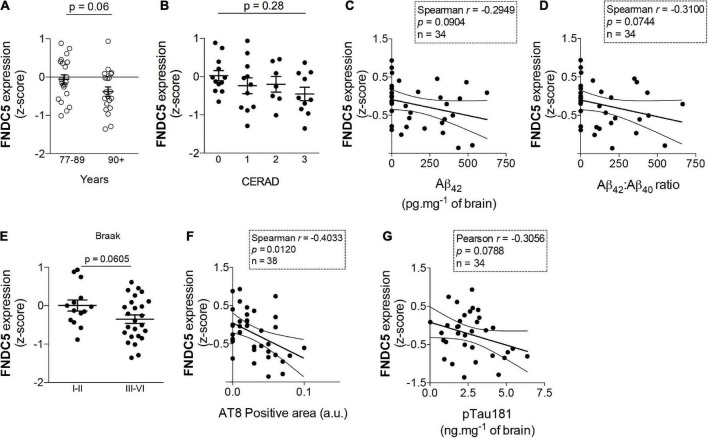
Fibronectin type III domain containing protein 5 (FNDC5) mRNA expression inversely correlates with age and AD neuropathology. Z-scores for FNDC5 expression obtained from the RNA seq database of the Adult Changes in Thought (ACT) study (https://aging.brain-map.org/). **(A)** FNDC5 expression in postmortem hippocampal tissue from 77 to 89 years old and 90 + year-old subjects, *N* = 21 cases of 77–89 years old and 20 cases of 90 + years old. **(B)** Hippocampal FNDC5 z-scores across the CERAD scale for amyloid pathology. (*N* = 12 for CERAD 0, 11 for CERAD 1, 7 for CERAD 2, and 12 for CERAD 3). Correlations between hippocampal FNDC5 z-scores and brain Aβ42 **(C)** or Aβ42/Aβ40 ratio (*N* = 34) **(D)**. **(E)** Hippocampal FNDC5 z-scores across the Braak scale for tau pathology. (*N* = 14 for Braak I-II and 25 for Braak III–VI). Correlations between hippocampal FNDC5 z-scores and brain AT8 (pSer202/Thr205 tau) (*N* = 38) **(F)** or pThr181 tau immunoreactivity (*N* = 34) **(G)**. Statistics: two-tailed unpaired Student’s *t*-test **(A,E)** or unpaired one-way ANOVA with Sidak *post-hoc* test **(B)**. Correlations **(C,D,F,G)** were determined using the Pearson method for parametric data or the Spearman method for non-parametric data.

As age is the major risk factor for AD-linked dementia, it is important to determine whether such endogenous mechanisms are disrupted in aging. To address this issue in humans, we analyzed the expression of hippocampal FNDC5 in data sets obtained from *postmortem* tissue from elderly subjects enrolled in the Adult Changes in Thought (ACT) study (see text footnote 1). We found that subjects older than 90 years old, who are at considerably high risk for AD and associated pathology (Corrada et al., 2010; Bullain and Corrada, 2013), present a trend for lower hippocampal expression of FNDC5 than individuals ranging from 77 to 89 years old (z-score mean difference: −0.32; *t* = 1.87; *p* = 0.06) (Figure 2A). We next investigated whether hippocampal FNDC5 mRNA levels were associated with AD-linked neuropathology in the studied cohort. While the expression of FNDC5 was not significantly altered across CERAD staging of amyloid pathology (CERAD 0: 0.02 ± 0.13; CERAD 1: −0.24 ± 0.20; CERAD 2: −0.20 ± 0.20; CERAD 2: −0.45 ± 0.18; *F* = 0.74; one-way ANOVA *p* = 0.06) (Figure 2B), we found trends of negative correlations between FNDC5 z-scores and brain Aβ_42_ level (Spearman *r* = 0.29; *p* = 0.09) (Figure 2C), and brain Aβ_42_/Aβ_40_ ratio (Spearman *r* = 0.31; *p* = 0.07) (Figure 2D). We further found that subjects with high tau pathology, as assessed by the Braak neuropathological scale (Braak and Braak, 1991), had a trend for reduced FNDC5 expression in the hippocampus (Braak I-II: 0.005 ± 0.14; Braak III-VI: −0.35 ± 0.11; *t* = 1.93; *p* = 0.06) (Figure 2E). Accordingly, reduced FNDC5 expression was associated with higher AT8-positive labeling (Spearman *r* = −0.40; *p* = 0.01) (Figure 2F), which reflects pSer202/Thr205 tau-positive neurofibrillary inclusions. FNDC5 z-scores also showed a trend of inverse association with pThr181-tau immunoreactivity in the hippocampus (Pearson *r* = −0.30; *p* = 0.07) (Figure 2G). Results thus raise the possibility that FNDC5 expression associates inversely with age and AD-related neuropathology (Aβ and tau) in the human hippocampus.

Recent studies suggest that irisin controls memory function (Lourenco et al., 2019, 2020; Jodeiri Farshbaf et al., 2020; Islam et al., 2021). The current study demonstrates that irisin signaling engages transient ERK phosphorylation (activation), increases extracellular BDNF, and prevents AβO-induced oxidative stress. Collectively, these observations expand our knowledge of how irisin signals in the brain and mediates the beneficial properties of physical exercise.

Of note, the temporal dynamics of ERK phosphorylation found here coincides with our previous demonstration of irisin-induced cAMP accumulation (Lourenco et al., 2019). Here we further demonstrate that cAMP accumulation by the adenylyl cyclase activator, forskolin, promotes ERK phosphorylation, raising the possibility that ERK activation is downstream of cAMP. Although aVβ5 integrin has been identified as a putative irisin receptor in the brain (Islam et al., 2021), the receptor(s) required for these effects induced by irisin in hippocampal neurons and the potential contribution of glial cells remain elusive. It is conceivable that irisin may exert pleiotropic actions in the brain by activating multiple signaling pathways in distinct brain cells (e.g., neurons and glial cells). Studies in other cell types indicate that irisin activates pathways mediated by p38MAPK, AMPK, and Akt (Zhang et al., 2014, 2020; Lin et al., 2021), but whether irisin stimulates the same pathways in the brain remains to be investigated.

Our results further suggest that hippocampal FNDC5 expression may correlate inversely with AD-linked neuropathology, in line with our previous reports that brain irisin is reduced in AD (Lourenco et al., 2019) and that CSF irisin correlates with memory and Aβ profiles typical of AD (Lourenco et al., 2020). Investigating FNDC5 mRNA levels in the human cohort allowed us to focus on local hippocampal FNDC5/irisin production and thus avoid potential confounders related to blood-brain barrier permeability to peripheral irisin or local irisin clearance. Nonetheless, future studies are warranted to determine whether aging or AD pathology can modify the dynamics of irisin production/clearance in the brain.

A limitation of the current study is the reduced sample size for the RNAseq datasets after application of the exclusion criteria we defined (TBI or non-Alzheimer’s dementia). Confirmation of these associations in larger cohorts will be important to extend the significance of the current findings. On the other hand, the parallel measurement of multiple Aβ and tau neuropathological markers, allowing examination of their associations with FNDC5 expression, represents an advantage of our study. Furthermore, our report of increased extracellular BDNF was based on commercial ELISA kits, which rely on antibody specificity and may be seen as another limitation. Replication of these findings using additional techniques (e.g., proteomics of cultured media) may confirm these results in future studies. Nonetheless, the current data are in line with previous evidence showing that transduction of primary neurons with AdFNDC5 promoted Fndc5 expression, as assessed by qPCR (Wrann et al., 2013). Further investigation of brain irisin signaling, including mechanistic studies, will be key to establish the potential of pharmacological therapeutics inspired by the protective actions of exercise in neurodegenerative diseases.

## Data availability statement

Publicly available datasets were analyzed in this study. This data can be found here: https://aging.brain-map.org/download/index.

## Ethics statement

The studies involving human participants were reviewed and approved by the Human data were obtained from public repositories of the Aging, Dementia and TBI study, for which appropriate approvals were obtained by the ACT study coordinators. More information can be found on https://help.brain-map.org/display/aging/Documentation. The patients/participants provided their written informed consent to participate in this study. The animal study was reviewed and approved by Institutional Animal Care and Use Committee of the Federal University of Rio de Janeiro (protocol # IBqM 022).

## Author contributions

ML, SF, and FD designed the study, analyzed and discussed the results, contributed the reagents, materials, animals, and analysis tools. ML, GF, and ÍR performed the research and analyzed data. All authors wrote the manuscript.

## Footnotes

1
https://aging.brain-map.org/

## Abbreviations

AD: Alzheimer’s disease
Aβ: amyloid- β
AβOs: amyloid- β oligomers
ACT: adult changes in thought study
BDNF: brain derived neurotrophic factor
cAMP: 3′,5′ -cyclic adenosine monophosphate
CERAD: consortium to establish a registry for Alzheimer’s disease
CREB: cAMP response element binding protein
ERK 1/2: extracellular signal-regulated kinase 1/2
FNDC5: fibronectin type III domain containing protein 5
GFP: green fluorescent protein
mRNA: messenger ribonucleic acid
PKA: cAMP-activated protein kinase
RNAseq: RNA sequencing
ROS: reactive oxygen species
TBI: traumatic brain injury.

## References

[B1] Abràmoff M. D. Magalhães P. J. Ram S. J. (2004). Image processing with ImageJ. *Biophotonics Int.* 11 36–42.

[B2] Almeida R. D. Manadas B. J. Melo C. V. Gomes J. R. Mendes C. S. Graos M. M. (2005). Neuroprotection by BDNF against glutamate-induced apoptotic cell death is mediated by ERK and PI3-kinase pathways. *Cell Death Differ.* 12 1329–1343. 15905876 10.1038/sj.cdd.4401662

[B3] Bomfim T. R. Forny-Germano L. Sathler L. B. Brito-Moreira J. Houzel J. C. Decker H. (2012). An anti-diabetes agent protects the mouse brain from defective insulin signaling caused by Alzheimer’s disease-associated Aβ oligomers. *J. Clin. Invest.* 122 1339–1353. 10.1172/JCI57256 22476196 PMC3314445

[B4] Boström P. Wu J. Jedrychowski M. P. Korde A. Ye L. Lo J. C. (2012). A PGC1-α-dependent myokine that drives brown-fat-like development of white fat and thermogenesis. *Nature* 481 463–468. 10.1038/nature10777 22237023 PMC3522098

[B5] Braak H. Braak E. (1991). Neuropathological stageing of Alzheimer-related changes. *Acta Neuropathol.* 82 239–259. 10.1007/BF00308809 1759558

[B6] Brito-Moreira J. Lourenco M. V. Oliveira M. M. Ribeiro F. C. Ledo J. H. Diniz L. P. (2017). Interaction of amyloid-beta (Abeta) oligomers with neurexin 2alpha and neuroligin 1 mediates synapse damage and memory loss in mice. *J. Biol. Chem.* 292 7327–7337. 10.1074/jbc.M116.761189 28283575 PMC5418035

[B7] Bullain S. S. Corrada M. M. (2013). Dementia in the oldest old. *Continuum* 19 457–469. 23558489 10.1212/01.CON.0000429172.27815.3f PMC4234050

[B8] Clarke J. R. Ribeiro F. C. Frozza R. L. De Felice F. G. Lourenco M. V. (2018). Metabolic defects in Alzheimer’s disease: From basic neurobiology to clinical approaches. *J. Alzheimers Dis.* 64 405–426. 29562518 10.3233/JAD-179911

[B9] Corrada M. M. Brookmeyer R. Paganini-Hill A. Berlau D. Kawas C. H. (2010). Dementia incidence continues to increase with age in the oldest old: The 90+ study. *Ann. Neurol.* 67 114–121. 20186856 10.1002/ana.21915 PMC3385995

[B10] De Bastiani M. A. Bellaver B. Carello-Collar G. Zimmermann M. Kunach P. Lima-Filho R. A. S. (2022). Transcriptomic similarities and differences between mouse models and human Alzheimer’s Disease. *bioRxiv* [Preprint]. 10.1101/2021.06.09.447404

[B11] De Felice F. G. Velasco P. T. Lambert M. P. Viola K. Fernandez S. J. Ferreira S. T. (2007). Aβ oligomers induce neuronal oxidative stress through an N-methyl-D-aspartate receptor-dependent mechanism that is blocked by the Alzheimer drug memantine. *J. Biol. Chem.* 282 11590–11601. 10.1074/jbc.M607483200 17308309

[B12] De Freitas G. B. Lourenco M. V. De Felice F. G. (2020). Protective actions of exercise-related FNDC5/Irisin in memory and Alzheimer’s disease. *J. Neurochem*. 155 602–611. 10.1111/jnc.15039 32396989

[B13] Freitas-Correa L. Lourenco M. V. Acquarone M. Da Costa R. F. Galina A. Rehen S. K. (2013). 2,4-Dinitrophenol induces neural differentiation of murine embryonic stem cells. *Stem Cell Res.* 11 1407–1416. 10.1016/j.scr.2013.09.016 24148244

[B14] Isaac A. R. Lima-Filho R. Lourenco M. V. (2021). How does the skeletal muscle contribute to brain function? *Neuropharmacology* 197:108744. 10.1016/j.neuropharm.2021.108744 34363812

[B15] Islam M. R. Valaris S. Young M. F. Haley E. B. Luo R. Bond S. F. (2021). Exercise hormone irisin is a critical regulator of cognitive function. *Nat. Metab.* 3 1058–1070. 34417591 10.1038/s42255-021-00438-z PMC10317538

[B16] Jedrychowski M. P. Wrann C. D. Paulo J. A. Gerber K. K. Szpyt J. Robinson M. M. (2015). Detection and quantitation of circulating human irisin by tandem mass spectrometry. *Cell Metab.* 22 734–740. 10.1016/j.cmet.2015.08.001 26278051 PMC4802359

[B17] Jodeiri Farshbaf M. Alvina K. (2021). Multiple Roles in Neuroprotection for the Exercise Derived Myokine Irisin. *Front. Aging Neurosci.* 13:649929. 10.3389/fnagi.2021.649929 33935687 PMC8086837

[B18] Jodeiri Farshbaf M. Garasia S. Moussoki D. P. K. Mondal A. K. Cherkowsky D. Manal N. (2020). Hippocampal injection of the exercise-induced myokine irisin suppresses acute stress-induced neurobehavioral impairment in a sex-dependent manner. *Behav. Neurosci.* 134 233–247. 10.1037/bne0000367 32437197

[B19] Kim H. Wrann C. D. Jedrychowski M. Vidoni S. Kitase Y. Nagano K. (2018). Irisin Mediates Effects on Bone and Fat via αV Integrin Receptors. *Cell* 175 1756–1768.e17. 30550785 10.1016/j.cell.2018.10.025 PMC6298040

[B20] Kirouac L. Rajic A. J. Cribbs D. H. Padmanabhan J. (2017). Activation of Ras-ERK Signaling and GSK-3 by Amyloid Precursor Protein and Amyloid Beta Facilitates Neurodegeneration in Alzheimer’s Disease. *eNeuro* 4 ENEURO.0149–16.2017. 10.1523/ENEURO.0149-16.2017 28374012 PMC5367084

[B21] Lin C. Guo Y. Xia Y. Li C. Xu X. Qi T. (2021). FNDC5/Irisin attenuates diabetic cardiomyopathy in a type 2 diabetes mouse model by activation of integrin alphaV/beta5-AKT signaling and reduction of oxidative/nitrosative stress. *J. Mol. Cell Cardiol.* 160 27–41. 10.1016/j.yjmcc.2021.06.013 34224725

[B22] Livak K. J. Schmittgen T. D. (2001). Analysis of relative gene expression data using real-time quantitative PCR and the 2(-Delta Delta C(T)) Method. *Methods* 25 402–408. 11846609 10.1006/meth.2001.1262

[B23] Lourenco M. V. Clarke J. R. Frozza R. L. Bomfim T. R. Forny-Germano L. Batista A. F. (2013). TNF-α mediates PKR-dependent memory impairment and brain IRS-1 inhibition induced by Alzheimer’s β-amyloid oligomers in mice and monkeys. *Cell Metab.* 18 831–843. 10.1016/j.cmet.2013.11.002 24315369

[B24] Lourenco M. V. Frozza R. L. De Freitas G. B. Zhang H. Kincheski G. C. Ribeiro F. C. (2019). Exercise-linked FNDC5/irisin rescues synaptic plasticity and memory defects in Alzheimer’s models. *Nat. Med.* 25 165–175. 30617325 10.1038/s41591-018-0275-4 PMC6327967

[B25] Lourenco M. V. Ribeiro F. C. Sudo F. K. Drummond C. Assuncao N. Vanderborght B. (2020). Cerebrospinal fluid irisin correlates with amyloid-beta, BDNF, and cognition in Alzheimer’s disease. *Alzheimers Dement.* 12:e12034. 10.1002/dad2.12034 32582833 PMC7306518

[B26] Moon H. S. Dincer F. Mantzoros C. S. (2013). Pharmacological concentrations of irisin increase cell proliferation without influencing markers of neurite outgrowth and synaptogenesis in mouse H19-7 hippocampal cell lines. *Metabolism* 62 1131–1136. 10.1016/j.metabol.2013.04.007 23664146 PMC4370428

[B27] Oliveira M. M. Lourenco M. V. Longo F. Kasica N. P. Yang W. Ureta G. (2021). Correction of eIF2-dependent defects in brain protein synthesis, synaptic plasticity, and memory in mouse models of Alzheimer’s disease. *Sci. Signal.* 14:eabc5429. 10.1126/scisignal.abc5429 33531382 PMC8317334

[B28] Pan J. A. Zhang H. Lin H. Gao L. Zhang H. L. Zhang J. F. (2021). Irisin ameliorates doxorubicin-induced cardiac perivascular fibrosis through inhibiting endothelial-to-mesenchymal transition by regulating ROS accumulation and autophagy disorder in endothelial cells. *Redox Biol.* 46:102120. 10.1016/j.redox.2021.102120 34479089 PMC8413906

[B29] Peng J. Deng X. Huang W. Yu J. H. Wang J. X. Wang J. P. (2017). Irisin protects against neuronal injury induced by oxygen-glucose deprivation in part depends on the inhibition of ROS-NLRP3 inflammatory signaling pathway. *Mol. Immunol.* 91 185–194. 10.1016/j.molimm.2017.09.014 28961497

[B30] Raony I. Domith I. Lourenco M. V. Paes-De-Carvalho R. Pandolfo P. (2022). Trace amine-associated receptor 1 modulates motor hyperactivity, cognition, and anxiety-like behavior in an animal model of ADHD. *Prog. Neuropsychopharmacol. Biol. Psychiatry* 117:110555. 10.1016/j.pnpbp.2022.110555 35346791

[B31] Saraiva L. M. Seixas Da Silva G. S. Galina A. Da-Silva W. S. Klein W. L. (2010). Amyloid-β triggers the release of neuronal hexokinase 1 from mitochondria. *PLoS One* 5:e15230. 10.1371/journal.pone.0015230 21179577 PMC3002973

[B32] Sebollela A. Freitas-Correa L. Oliveira F. F. Paula-Lima A. C. Saraiva L. M. Martins S. M. (2012). Amyloid-β oligomers induce differential gene expression in adult human brain slices. *J. Biol. Chem.* 287 7436–7445. 10.1074/jbc.M111.298471 22235132 PMC3293600

[B33] Smith M. A. Hirai K. Hsiao K. Pappolla M. A. Harris P. L. Siedlak S. L. (1998). Amyloid-beta deposition in Alzheimer transgenic mice is associated with oxidative stress. *J. Neurochem.* 70 2212–2215. 9572310 10.1046/j.1471-4159.1998.70052212.x

[B34] Wrann C. D. White J. P. Salogiannnis J. Laznik-Bogoslavski D. Wu J. Ma D. (2013). Exercise Induces Hippocampal BDNF through a PGC-1α/FNDC5 Pathway. *Cell Metab.* 18 649–659. 24120943 10.1016/j.cmet.2013.09.008 PMC3980968

[B35] Zhang X. Hu C. Kong C. Y. Song P. Wu H. M. Xu S. C. (2020). FNDC5 alleviates oxidative stress and cardiomyocyte apoptosis in doxorubicin-induced cardiotoxicity via activating AKT. *Cell Death Differ.* 27 540–555. 10.1038/s41418-019-0372-z 31209361 PMC7206111

[B36] Zhang Y. Li R. Meng Y. Li S. Donelan W. Zhao Y. (2014). Irisin stimulates browning of white adipocytes through mitogen-activated protein kinase p38 MAP kinase and ERK MAP kinase signaling. *Diabetes* 63 514–525. 10.2337/db13-1106 24150604 PMC13117908

