# Supplementary material for: Irisin stimulates protective signaling pathways in rat hippocampal neurons
Source: Front Cell Neurosci. 2022 Sep 9;16:953991. doi: 10.3389/fncel.2022.953991 (PMC9518673; doi:10.3389/fncel.2022.953991)
Supplement: Supplementary file 1 [file Data_Sheet_1.pdf]

Supplementary Material

Irisin stimulates protective signaling pathways in rat hippocampal neurons

Mychael V. Lourenco[1*], Guilherme B. de Freitas[1,2], Ícaro Raony[1],

Sergio T. Ferreira[1,3*], Fernanda G. De Felice[1,2,4*]

[1]Institute of Medical Biochemistry Leopoldo de Meis, Federal University of Rio de Janeiro, Rio de Janeiro, RJ, Brazil

[2]Centre for Neuroscience Studies, Departments of Biomedical and Molecular Sciences & Psychiatry, Queen's University, Kingston, ON, Canada

[3]Institute of Biophysics Carlos Chagas Filho, Federal University of Rio de Janeiro, Rio de Janeiro, RJ, Brazil.

[4]D'Or Institute for Research and Education, Rio de Janeiro, RJ, Brazil

*Corresponding authors: Fernanda G. De Felice (fernanda.defelice@queensu.ca), Sergio T. Ferreira (ferreira@bioqmed.ufrj.br) and Mychael V. Lourenco (mychael@bioqmed.ufrj.br).

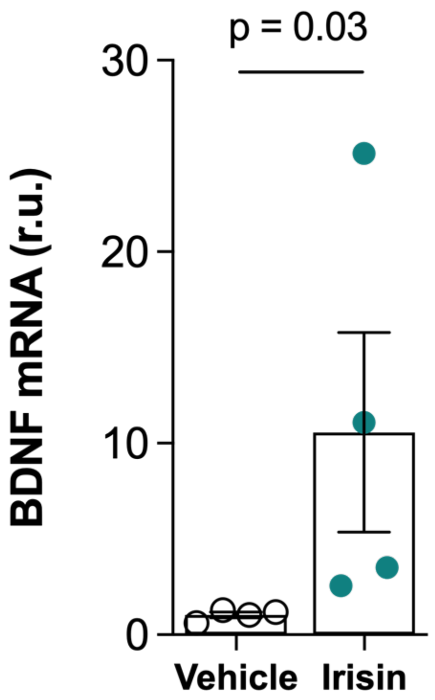

Supplementary Figure 1. Treatment with recombinant irisin increases BDNF expression in primary rat hippocampal cultures. BDNF expression (measured by qRT-PCR) in rat neuronal cultures treated with 25 nM irisin or vehicle for 24 h. Data were normalized by β-actin. $p = 0.03$, two-tailed ratio paired T-test. N = 4 independent cultures. Each dot represents an independent experiment.
